# Estrogen Receptor β Exerts Tumor Repressive Functions in Human Malignant Pleural Mesothelioma via EGFR Inactivation and Affects Response to Gefitinib

**DOI:** 10.1371/journal.pone.0014110

**Published:** 2010-11-29

**Authors:** Giulia Pinton, Warren Thomas, Paolo Bellini, Arcangela Gabriella Manente, Roberto E. Favoni, Brian J. Harvey, Luciano Mutti, Laura Moro

**Affiliations:** 1 Department of Chemical, Food, Pharmaceutical and Pharmacological Sciences, Drug and Food Biotechnology Center, University of Piemonte Orientale A. Avogadro, Novara, Italy; 2 Department of Molecular Medicine, Royal College of Surgeons in Ireland and Education and Research Centre, Beaumont Hospital, Dublin, Ireland; 3 Laboratory of Experimental Pharmacology, National Cancer Institute, Genoa, Italy; 4 Department of Medicine, Local Health Unit 11, Vercelli, Italy; Bauer Research Foundation, United States of America

## Abstract

**Background:**

The role of estrogen and estrogen receptors in oncogenesis has been investigated in various malignancies. Recently our group identified estrogen receptor beta (ERβ) expression as an independent prognostic factor in the progression of human Malignant Pleural Mesothelioma (MMe), but the underlying mechanism by which ERβ expression in tumors determines clinical outcome remains largely unknown. This study is aimed at investigating the molecular mechanisms of ERβ action in MMe cells and disclosing the potential translational implications of these results.

**Methods:**

We modulated ERβ expression in REN and MSTO-211H MMe cell lines and evaluated cell proliferation and EGF receptor (EGFR) activation.

**Results:**

Our data indicate that ERβ knockdown in ER positive cells confers a more invasive phenotype, increases anchorage independent proliferation and elevates the constitutive activation of EGFR-coupled signal transduction pathways. Conversely, re-expression of ERβ in ER negative cells confers a more epithelioid phenotype, decreases their capacity for anchorage independent growth and down-modulates proliferative signal transduction pathways. We identify a physical interaction between ERβ, EGFR and caveolin 1 that results in an altered internalization and in a selective reduced activation of EGFR-coupled signaling, when ERβ is over-expressed. We also demonstrate that differential expression of ERβ influences MMe tumor cell responsiveness to the therapeutic agent: Gefitinib.

**Conclusions:**

This study describes a role for ERβ in the modulation of cell proliferation and EGFR activation and provides a rationale to facilitate the targeting of a subgroup of MMe patients who would benefit most from therapy with Gefitinib alone or in combination with Akt inhibitors.

## Introduction

Malignant pleural mesothelioma (MMe) is a highly aggressive tumor, most often associated with asbestos exposure, although a role for SV40 and genetic susceptibility have also been proposed [1]. The delayed clinical diagnosis of this tumor is due to the slow progression of the malignancy [2]. The clinical prognosis is generally poor, with a reported median survival from presentation of 9–12 months. Several clinical prognostic factors have been tentatively correlated to patient survival; these include histological type (epithelioid, sarcomatoid or biphasic) and tumor grade [3], [4]. We recently published data demonstrating that estrogen receptor beta (ERβ) is linked with better prognosis in MMe patients and is likely to act as tumor repressor [5].

Estrogens exert their biological effects through two distinct receptors: ERα and ERβ. The ERs are transcribed from two different genes and display specific tissue expression patterns as well as distinct ligand specificities even though both bind the most biologically active estrogen, 17β-estradiol [6]. This is confirmed by the fact that mice lacking ERβ (βER KO) display a very different phenotype to those devoid of ERα (αERKO) [7]–[11]. In addition to ligand binding ERβ activity and sub-cellular distribution is also regulated through its post-translational modification [12], [13]. Evidences accumulated over the past decade describe a cross-talk between ERs and EGFRs [14]. Work in this area has established a requirement of ERs for some EGFR actions [15], [16]. Recent findings suggest the important role of EGFR (or similar receptors) for estrogen signaling from the membrane in breast cancer. It has been shown that a pool of ERα resides in or associates with the plasma membrane and utilizes the membrane EGFR to rapidly signal through various kinase cascades that influence both transcriptional and non-transcriptional actions of estrogen in breast cancer cells [14], [17]. Moreover, the activation of ERK1/2 through EGFRs and IGFR changes the phosphorylation state of ERα to modulate receptor localization and transcriptional activity [18], [19]. More recently, it has become clear that ERβ function can also be modulated by phosphorylation in its N-terminal region, so coupling ERβ activity to growth factor signaling [20].

A large number of studies have focused on the expression of growth factor receptors in MMe. EGFR is over-expressed in MMe and this correlates significantly with increased tumor cell proliferation and with the promotion of angiogenesis [21], [22]. Despite these evidences two phase II studies with Erlotinib and Gefitinib, two anti-phospho tyrosine kinase EGFR specific molecules, did not show efficacy suggesting that further characteristic apart from EGFR expression could be involved in determining sensitivity to these agents [23], [24].

The aim of this study is to achieve a better knowledge on the molecular mechanism by which ERβ exerts its tumor repressor effects on MMe progression, in view of potential novel patient-tailored therapies.

## Results

### ERβ expression in ERs negative MMe cells reduces their growth rate

**Figure 1:**
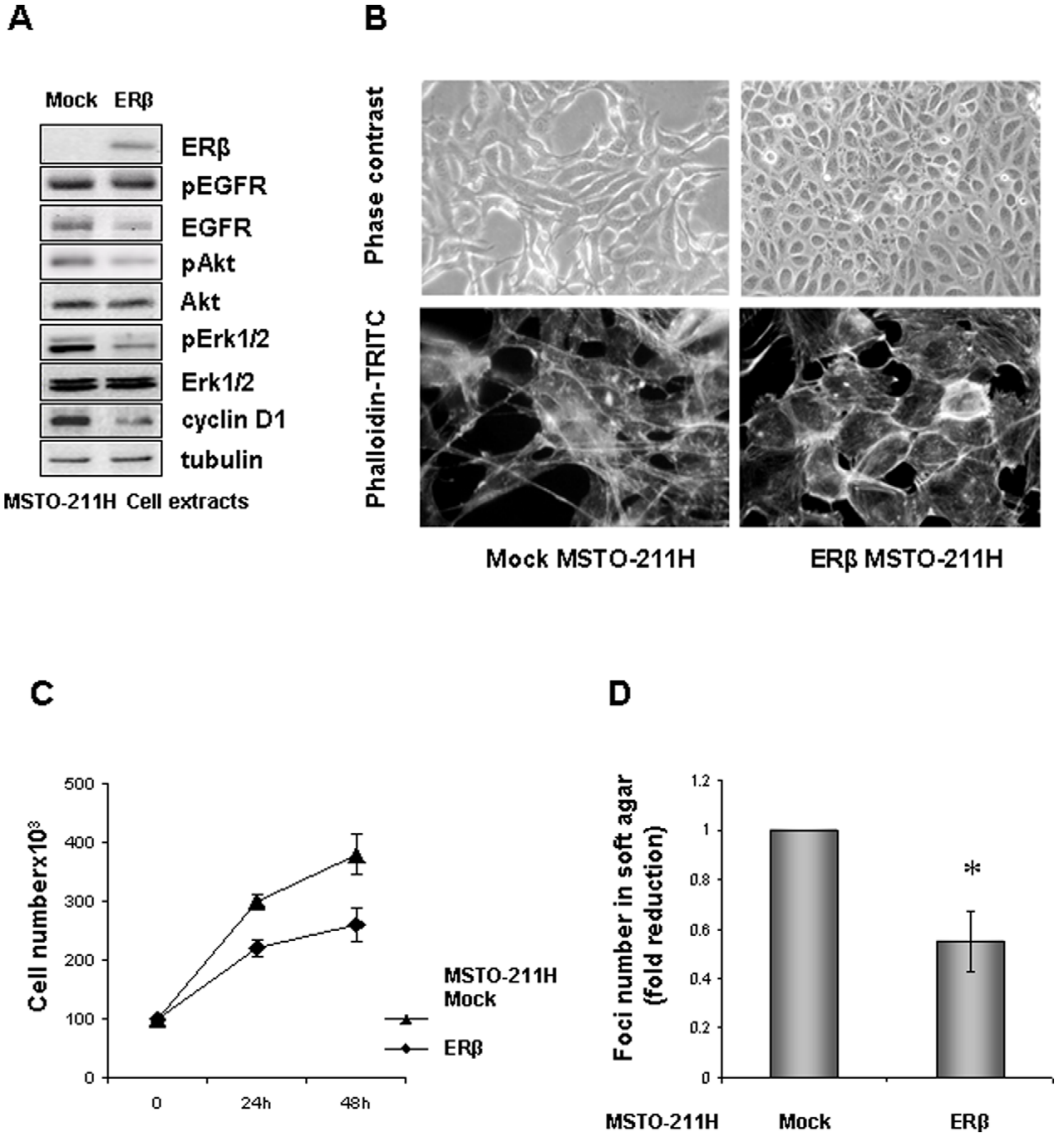
ERβ expression in ERs negative MMe cells reduces their growth rate. **A**) Western Blot analysis of cell extracts from mock- and ERβ expressing MSTO-211H cells. Representative of three separate experiments. **B**) Upper panels show phase contrast microphotographs (200X magnification) of mock- or ERβ-transfected MSTO-211H cells, visualizing the acquisition of a more epithelioid phenotype in transfected cells. Lower panels show cells fixed in ethanol and stained for actin with phalloidin–TRITC as described. Note the actin rearrangement in ERβ expressing cells (400X magnification). **C**) Cell proliferation curves of mock- and ERβ-transfected MSTO-211H cells cultured in complete medium for 24 and 48 hours. Each value represents mean ± SD (n = 3). **D**) Total soft agar colony counts for mock- or ERβ-transfected MSTO-211H cells were done by three independent investigators microscopically visualizing individual colonies (clusters of 15 or more cells) in 10 random microscopic fields. Columns represent the fold increase of the mean number of colonies in 10 fields; bars, SD; * p<0.05. Representative of three separate experiments.

To confirm the tumor repressor role of ERβ in the modulation of MMe cell growth, we expressed ERβ in the constitutively ERs-negative MSTO-211H MMe cell line, by using a pCXN2 based plasmid expressing ERβ. ERβ expression conferred a more epithelioid phenotype on the MSTO-211H cells compared to mock transfected cells, characterized by a more cortical actin distribution (Fig. 1B). 48 hours after transfection, total protein extracts were prepared from mock- and ERβ -transfected cells. Equal amounts of protein from these cell extracts were Western blotted and probed with ERβ, phospho-EGFR, EGFR, phospho-Akt, Akt, phospho-ERK1/2, ERK1/2 and cyclin D1-specific antibodies; tubulin was added to confirm equal loading (Fig. 1A). ERβ protein expression was not detectable by immunoblot in mock transfected cells, whereas it was in transfected cells. Western blot analysis confirmed the efficacy of ERβ expression to down modulate EGFR, Akt and ERK1/2 phosphorylation without a change in the total abundance of these proteins expression, while it resulted in cyclin D1 protein reduction (Fig. 1A). Consistent with our published data [5], ERβ expression exerted at any considered time a significant (p<0.05) suppressive effect on MSTO-211H cell proliferation (Fig. 1C), without inducing apoptosis (data not shown); we believe that in these cells PI3K/Akt could act in concert with MAP kinase signaling to modulate cyclin D1 expression and cell cycle progression. Here we found that ERβ expression significantly reduced the number and the size of colonies that MSTO-211H cells formed when cultured for 7 days in soft agar. When colonies of more than 15 cells were considered about 50% of reduction was seen in ERβ expressing cells (Fig. 1D).

### ERβ silencing promotes MMe cell proliferation

**Figure 2:**
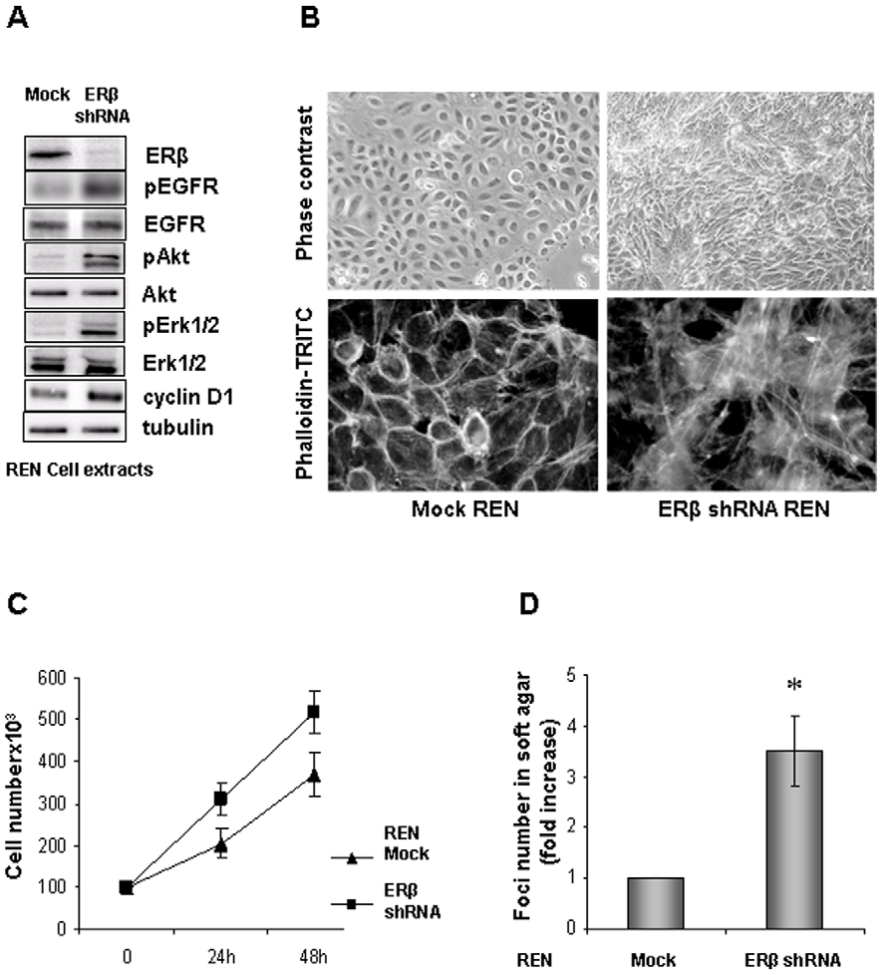
ERβ silencing promotes MMe cell proliferation. **A**) Western Blot analysis of cell extracts from mock- and ERβ silenced REN cells. Representative of three separate experiments. **B**) Upper panels show phase contrast microphotographs (200X magnification) of mock- or shERβ-transfected REN cells, visualizing the loss of contact inhibition and formation of foci *in vitro*. Lower panels show cells fixed in ethanol and stained with phalloidin-TRITC to stain for actin as described. Note the actin rearrangement in ERβ silenced cells (400X magnification). **C**) Cell proliferation curves of mock- and shERβ-transfected REN cells cultured in complete medium for 24 and 48 hours. Each value represents mean ± SD (n = 3). **D**) Total soft agar colony counts for mock- or shERβ-transfected REN cells were done by three independent investigators microscopically visualizing individual colonies (clusters of 15 or more cells) in 10 random microscopic fields. Columns represent the fold increase of the mean number of colonies; bars, SD; * p<0.05. Representative of three separate experiments.

We tested whether the suppression of ERβ expression could influence the rate of MMe cell proliferation. We previously established that REN cells express moderate levels of ERβ [5]. REN cells were transfected with an ERβ-specific shRNA (shRNA- ERβ) to suppress expression of the receptor. 48 hours after transfection, total protein extracts were prepared from mock- or shRNA-ERβ-transfected cells. Equal amounts of protein from these cell extracts were Western blotted and probed with ERβ, phospho-EGFR, EGFR, phospho-Akt, Akt, phospho-ERK1/2, ERK1/2 and cyclin D1-specific antibodies; tubulin was added to confirm equal loading (Fig. 2A). Western blot analysis confirmed the efficacy of the ERβ-specific shRNA in suppressing expression of the protein. Silencing of ERβ expression in REN cells resulted in increased EGFR, Akt and ERK1/2 phosphorylation without a change in the total abundance of the proteins. However, the abundance of cyclin D1 protein was elevated in cells suppressed in ERβ expression. Phase contrast microscopy imaging of cells grown on a solid substrate (Fig. 2B) revealed that silencing of ERβ resulted in the loss of contact inhibition by the REN cells, which allowed them to form dense foci rather than a confluent monolayer. The cells were fixed and stained with phalloidin-TRITC to discriminate changes in the actin cytoskeleton of the REN cells. Suppression of ERβ resulted in significant remodeling of the actin structure within the REN cells, with a transition from a largely cortical actin polymerization pattern to a highly defined stress fibers organization. We next performed cell proliferation experiments on cells grown on a solid surface and also tested the effect of ERβ suppression on the capacity of the REN cells for anchorage-independent growth in semi-solid media. ERβ suppression significantly (p<0.05) increased the proliferation rate of REN cells compared to wild-type controls at any considered time (Fig. 2C). Moreover, ERβ suppression resulted also in a 3 to 4-fold increase in the number of colonies formed by the REN cells after 7 days of culture in soft agar (Fig. 2D).

### ERβ over-expression influences EGFR mediated signaling and internalization

**Figure 3:**
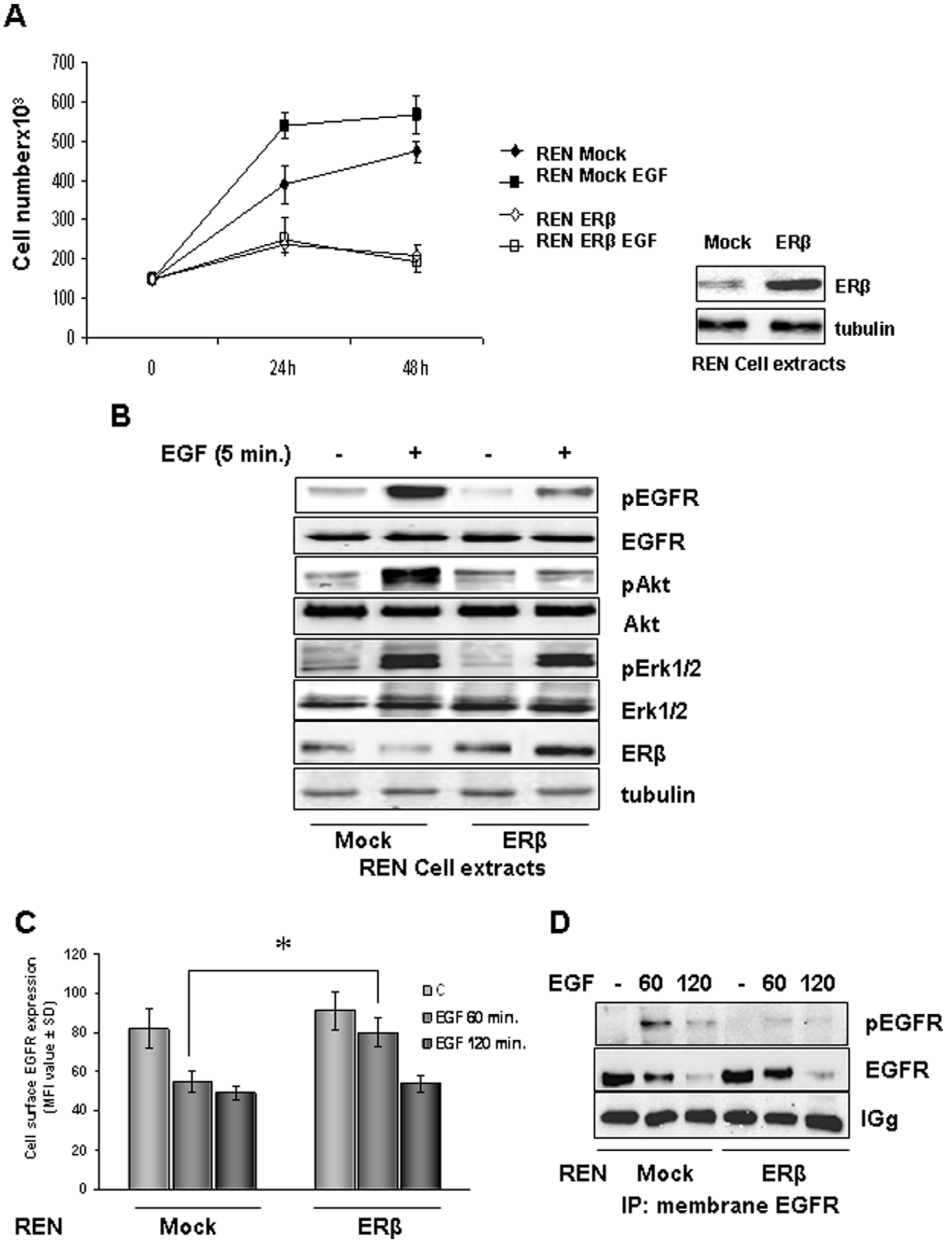
ERβ over-expression influences EGFR mediated signaling and internalization. **A**) The graph show the growth curves of mock- and ERβ-transfected REN cell treated for 24 and 48 hours with 5 ng/ml of EGF in 2% FBS culture medium. At each time point, the cells were assayed for proliferation. Each value represents mean ± SD (n = 3). Adjacent to the graph is reported a representative Western blot analysis that documents ERβ expression. Tubulin staining indicates equal loading of the proteins. **B**) Mock- and ERβ- transfected REN cells made quiescent for 2 hours were treated with 5 ng/ml of EGF for 5 minutes and detergent extracted. Levels of phosphorylated EGFR, ERK 1/2 MAP kinases and Akt were analyzed by immunoblotting. Membranes were also blotted with antibodies to EGFR, Erk1/2 and Akt to evaluate protein expression. Tubulin was blotted to show equal amount of loading. Western blot analysis with anti ERβ antibodies documents its expression in transfected cells. Representative of three separate experiments. **C**) Evaluation of EGFR internalization was performed by Flow cytometry analysis on wild type and ERβ expressing REN cells treated 60 or 120 minutes with 10 ng/ml of human recombinant EGF. Histograms represent percentage of positive cells following incubation with anti-EGFR antibody indicated for each condition ± SD. Data are representative of three separate experiments. **D**) Representative immunoprecipitation experiment of membrane associated EGFR performed on mock and ERβ over-expressing REN cells, treated 60 or 120 minutes with 10 ng/ml of human recombinant EGF. Membrane was blotted with anti-pY and anti-EGFR antibodies.

**Figure 4:**
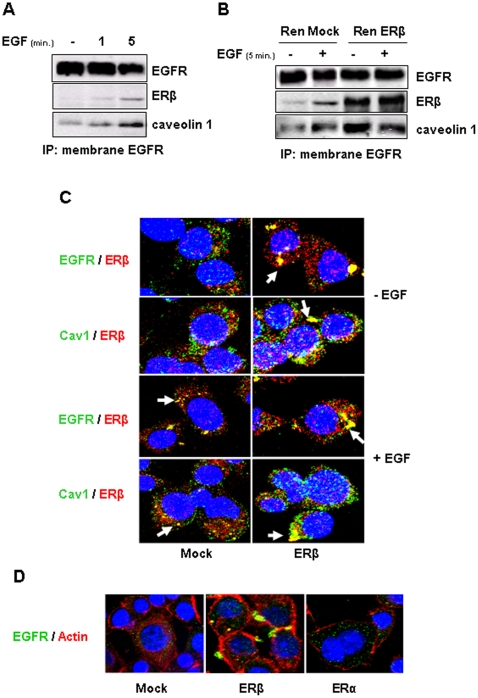
ERβ associates with EGFR and caveolin 1. **A**) Co-immunoprecipitation experiments were performed on REN cells treated 1 and 5 minutes with 5 ng/ml of human recombinant EGF. ERβ and caveolin 1 were detected by Western blot in immunoprecipitations of membrane associated EGFR. **B**) Co-immunoprecipitation experiments were performed on mock and ERβ over-expressing REN cells treated 5 minutes with 5 ng/ml of human recombinant EGF. ERβ and caveolin 1 were detected by Western blot in immunoprecipitations of membrane associated EGFR. **C**) Confocal double fluorescent microscopy analysis of red-labeled ERβ with green-labeled EGFR or caveolin 1 in mock- (left panel) or ERβ-transfected (right panel) REN cells treated or not 5 minutes with 5 ng/ml of human recombinant EGF. **D**) Confocal fluorescent microscopy analysis showing the localization of green-labeled EGFR and phalloidin-TRITC labeled actin filaments in mock and in ERβ and ERα transfected REN cells. Nuclei were counterstained with DAPI.

Therefore, we sought to investigate EGFR signaling in mock- and ERβ-transfected REN cells treated with EGF. Here we show that the proliferation of REN cells is promoted by EGF treatment; while transfection of REN cells with the ERβ expression plasmid significantly (p<0.05) inhibited the proliferation rate of these cells both under basal conditions and following EGF exposure (Fig. 3A). In response to EGF treatment of mock cells, EGFR became phosphorylated and the ERK1/2 MAPK and Akt signaling pathways were activated as demonstrated by the phosphorylation state of these kinases (Fig. 3B). In ERβ over-expressing cells there was a reduced basal level of EGFR phosphorylation and a diminished response to EGF treatment. This translated into a reduced activation of signal transduction cascades with a slight reduction in EGF induced ERK1/2 phosphorylation, but a complete ablation of the EGF induced Akt phosphorylation (Fig. 3B). To assess whether EGFR internalization was affected by ERβ over-expression, we evaluated the process of EGFR internalization at 60 and 120 minutes of EGF treatment, in mock and ERβ over-expressing REN cells. As shown in Fig. 3C, EGFR is almost completely internalized in mock-REN cells at both 60 and 120 minutes. In ERβ-REN cells, EGFR is internalized at both time points, although the process appears to be slower with respect to mock cells, in particular at 60 minutes, suggesting a different kinetic of internalization/recycling. These data were confirmed by immunoblot analysis with anti-phospho tyrosine and anti-EGFR antibodies of EGFR immunoprecipitated from plasma membrane (Fig. 4D) of EGF treated mock and ERβ over-expressing REN cells.

### ERβ associates with EGFR and caveolin 1

Recently, it has been shown that EGFR may also follow the two distinct endocytotic routes: one clathrin-dependent and one clathrin-independent mediated by caveolin [25]. Published evidence suggests that the EGFR-caveolin interaction leads to reduced activation of EGFR signaling [26]. The interaction between androgen receptor and EGFR in the caveolae of prostate cancer cells has been recently reported [27]. Consequently, we investigated the physical interaction between ERβ, EGFR and caveolin-1 in REN cells. Firstly, membrane associated EGFR was immunoprecipitated from lysates of REN cells treated 1 and 5 minutes with 5 ng/ml of human recombinant EGF. As shown in Fig. 4A, Western blot analysis evidenced increased amounts of ERβ and caveolin 1 in EGFR immunoprecipitates upon EGF stimuli. Membrane-associated EGFR was then immunoprecipitated from mock- and ERβ -transfected REN cells that were treated for 5 minutes with EGF or left untreated, and then analyzed by immunoblotting with EGFR, ERβ and caveolin-1 antibodies. ERβ and caveolin-1 co-immunoprecipitated with EGFR to a minor extent in untreated cells however, EGF treatment promoted the interaction and more ERβ and caveolin-1 was immunoprecipitated with EGFR. Over-expression of ERβ resulted in an increased and EGF-independent association of these proteins (Fig 4B). The ERβ-EGFR-caveolin 1 interaction was further investigated by confocal imaging in mock-transfected and in ERβ over-expressing REN cells (Fig. 4C). In mock-transfected cells, there was little co-localization of ERβ with either EGFR or caveolin 1 within the cytoplasm or at the cell membrane; EGF treatment resulted in a co-localization of ERβ with EGFR and caveolin 1 at discrete sites largely located within the cytoplasm of treated cells. In ERβ over-expressing REN cells ERβ was associated at high abundance with EGFR and caveolin 1 at discrete sites within the cytoplasm, proximal to the cell membrane, supporting co-immunoprecipitation data. The co-localization of the proteins occurred independently of EGF treatment. The redistribution of EGFR to discrete sites was specific to over-expression of ERβ and was not observed when ERα was over-expressed in these cells (Fig. 4D).

### ERβ expression influences response of MMe cells to Gefitinib

**Figure 5:**
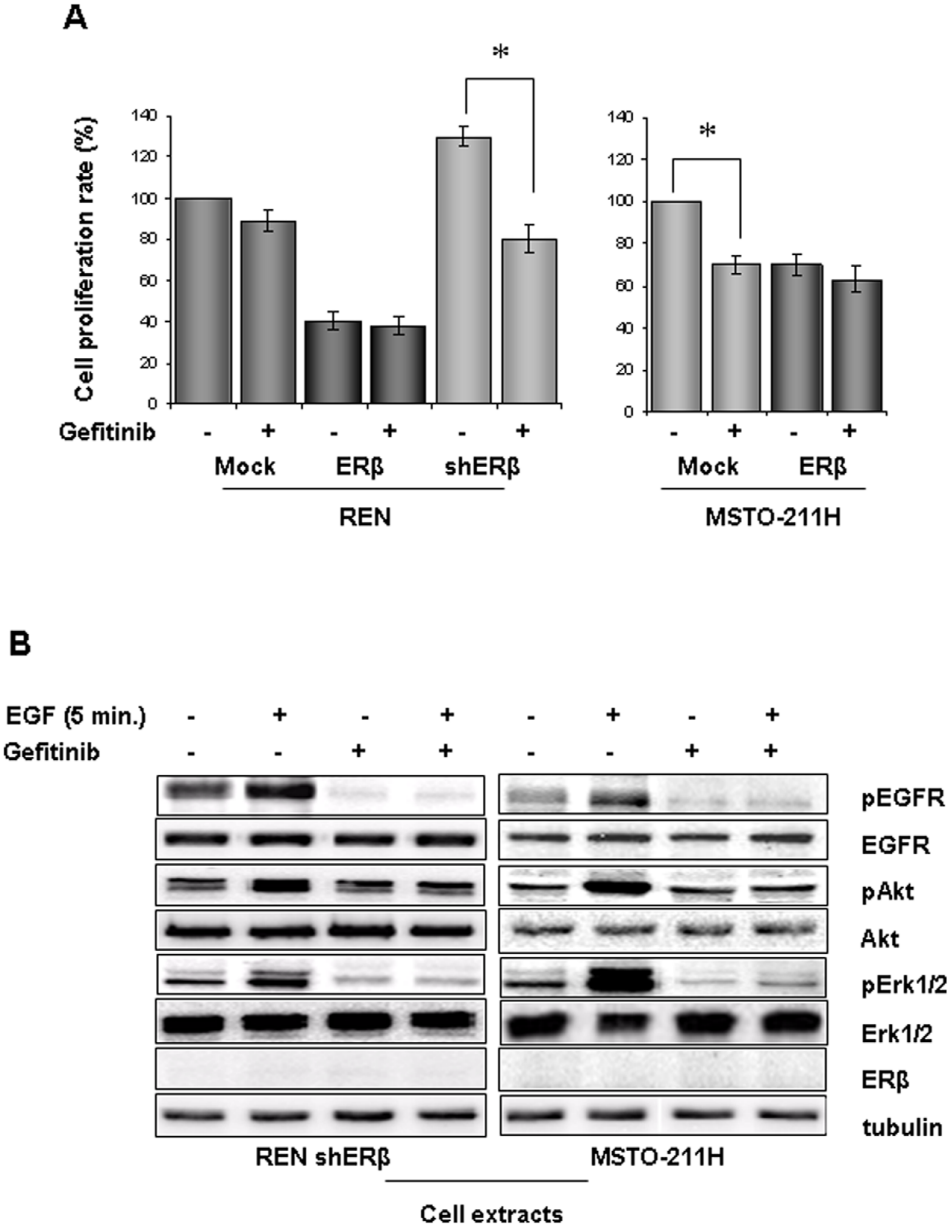
ERβ expression influences response of MMe cells to Gefitinib. **A**) Effects of Gefitinib on viable number were evaluated in mock-, ERβ- and shERβ-transfected REN and in mock- and ERβ- transfected MSTO-211H cell lines. Cells were incubated in serum-containing medium in the presence of 5 µM Gefitinib for 24–48 hours. As control 0.1% DMSO vehicle alone was used. Results are expressed as number of viable cells relative to control at 48 hours of treatment; bars, ± SD; * p<0.05. Data are representative of three separate experiments. **B**) shERβ-transfected REN and MSTO-211H cells were treated with 5 ng/ml of EGF for 5 minutes in the absence or presence of 5 µM Gefitinib and detergent extracted. Levels of phosphorylated EGFR, ERK 1/2 MAP kinases and Akt were analyzed by immunoblot. Membranes were also blotted with antibodies to EGFR, Erk1/2, and Akt to evaluate protein expression. Tubulin was blotted to show equal amount of loading. Western blot analysis with anti ERβ antibodies documents expression in transfected cells. Representative of three separate experiments.

Gefitinib is an EGFR tyrosine kinase inhibitor that acts by binding to the adenosine triphosphate (ATP)-binding site of the enzyme, employed in the treatment of certain types of carcinomas. However, lack of correlation between EGFR expression and response to its tyrosine-kinase (TK) inhibitor Gefitinib has been reported in different malignancies [28], [29]. Mutations in EGFR-TK domain have been associated with response in patients with metastatic NSCLC [30]. The prevalence of such mutations in mesothelioma is presently unknown but it seems that they are very rare in mesothelioma [31]. Here we tested if ERβ expression could influence response to Gefitinib of MMe cells. The growth-inhibitory effects of 5 µM Gefitinib were evaluated on mock, ERβ over-expressing or ERβ silenced REN cells and in mock and ERβ expressing MSTO-211H cells (Fig. 5A). REN cells were weakly sensitive to Gefitinib, and over-expression of ERβ did not significantly affect the sensitivity of these cells. The silencing of ERβ expression rendered the cells more sensitive to EGFR antagonism, suggesting that the loss of ERβ expression resulted in a greater reliance of the cells upon EGFR-coupled signaling pathways to support proliferation. These data were reinforced by concordant data obtained with Gefitinib treatment of wild type and ERβ positive MSTO-211H cells (Fig. 5A).

Because EGFR signals through PI3-kinase/Akt and MAP/ERK effectors pathways, phosphorylation of Akt and ERK 1/2 were analyzed in ERβ silenced REN and in MSTO-211H cells treated with EGF in the absence or in the presence of Gefitinib. The basal level of phosphorylated EGFR was increased in both cell types upon EGF treatment and this resulted in increased Akt and ERK1/2 phosphorylation. Gefitinib addition abrogated both basal and EGF induced EGFR and ERK1/2 phosphorylation, but only the EGF induced amount of phosphorylated Akt (Fig. 5B).

## Discussion

ERα and ERβ act as ligand-regulated transcription factors that positively and negatively regulate gene expression, directly or indirectly, through the activation of protein kinase signaling. Models of action have been described that involve synergism, as well as competition between the two receptors, which is further refined by other transcription factor interactions [32]. ERα and ERβ display differential expression profiles in estrogen-responsive tissues [33] and shifts in their expression profile have also been identified in malignant as compared to normal tissue and also through the stages of cancer progression [34]. We have found that ERβ is the predominant isoform expressed in the pleural mesothelial cells and patients with ERβ over expressing tumors have a better survival [5]. Evidence points to ERβ having a significant anti-neoplastic role in MMe but the mechanisms underpinning this role remain to be elucidated. In the present study we transfected the ERβ-expressing REN MMe cell line with ERβ-specific shRNA to suppress expression. This resulted in the promotion of anchorage-independent cell growth and transition to a less epithelioid phenotype. The mechanisms responsible for the increased cell growth and the phenotypic shift that correlates with the loss of ERβ expression are important in understanding the role of ERβ as a tumor suppressor. Conversely, exogenous expression of ERβ by the ERβ-negative, MSTO-211H MMe cell line resulted in suppression of anchorage-independent cell growth and transition to a more epithelioid phenotype. We previously demonstrated that *in vitro* ERβ over-expression caused a G2/M cell cycle phase arrest of MMe cells, both in a ligand-dependent and -independent manner. The fact that exogenous expression of ERβ leads to inhibition of proliferation correlates with *in vivo* data showing that ERβ expression was lost in the more aggressive sarcomatoid forms of the malignancy. In this present study we found that MMe cells silenced or constitutively devoid of ERβ expression also display a more aggressive phenotype, with the enhanced formation of foci when cultured *in vitro* and the development of more abundant colonies when cultured in soft agar.

The modulation of cell cycle regulating proteins through ERβ is compatible with rapidly induced signaling and ablation of ERβ impacts upon the activation but not the expression of multiple signaling intermediates in the MMe cells including Akt and Erk1/2. Cross-talk between ERs and growth factor receptor-mediated pathways at the plasma membrane has been described [35]–[37] and functional interactions between ERβ and the epidermal growth factor receptor (EGFR) is documented [20]. Over expression of EGFR has been detected in up to 68% of MMe tumors, however, the EGFR expression level is itself not a good prognostic indicator. In the present study we investigated the interaction between EGFR and ERβ in MMe cells. In cells which express high levels of ERs, ERβ but not ERα constitutively co-localizes with EGFR in caveolin 1 enriched regions. This clustering interferes with EGFR phosphorylation in response to its ligand, and also results in delayed internalization of the receptor and activation of coupled signaling cascades following stimulation. As a consequence, ERβ over-expressing cells are insensitive to treatment with the EGFR inhibitor, Gefitinib, while cells silenced in ERβ expression display basal EGFR phosphorylation and are more sensitive to Gefitinib. Our data give a possible explanation for the inefficacy of EGFR inhibitors in phase II clinical trials for ERβ positive epithelioid MMe patients and opens the possibility of a more successful employment of these drugs in more aggressive, ERβ negative, tumors either as a single agent or in combination with Akt inhibitors.

## Materials and Methods

### Reagents and antibodies

The monoclonal antibody specific for α-tubulin and the polyclonal antibodies for ERα,ERβ, EGFR, caveolin-1, ERK1/2 MAP kinase and cyclin D1 were from Santa Cruz Biotechnology (Santa Cruz, CA). The monoclonal antibody specific for Akt and the phosphorylation site-specific polyclonal antibodies for ERK1 (pThr202 and pTyr204), ERK2 (pThr185 and pTyr187) MAP kinases, and Akt (pSer473 and pThr308) were from Cell Signaling Technology (Beverly, MA). The polyclonal antibodies for EGFR and ERβ, used in immunofluorescence analysis were obtained from Calbiochem (Darmstadt, Germany) and Zymed-Invitrogen (Carlsbad, CA), respectively. Protein A-Sepharose and ECL were from Amersham Pharmacia Biotech (Uppsala, Sweden). Nitrocellulose membranes and protein assay kits were from Bio-Rad (Hercules, CA). The polyclonal phosphorylation site-specific antibody for EGFR (pTyr1086), anti-mouse and anti-rabbit IgG horseradish peroxidase conjugated antibodies, human recombinant EGF and all other chemical reagents unless otherwise specified were from Sigma-Aldrich (St Louis, MO). All reagents were of analytical grade. Culture media, sera, antibiotics, and LipofectAMINE were from Invitrogen (Carlsbad, CA). Gefitinib is an EGFR inhibitor also used clinically as a chemotherapeutic agent and it is marketed by AstraZeneca.

### Cell cultures treatments and transfection

The epithelioid MMe derived REN cell line that was used as the principal experimental model in this investigation was isolated, characterized [38] and kindly provided by Dr. S.M. Albelda (University of Pennsylvania, Philadelphia, PA) and the MSTO-211H cell line established from the pleural effusion of a patient with biphasic mesothelioma of the lung [39] was obtained from the *Istituto Scientifico Tumori* (IST)-Cell-bank, Genoa, Italy. Cells were cultured in RPMI medium supplemented with 10% foetal bovine serum (FBS) at 37°C in a 5% CO_2_-humidified atmosphere. For experimental purposes, the cells were maintained in the same culture medium but lacking phenol red and containing charcoal-stripped FBS. Mycoplasma infection was excluded by the use of the Mycoplasma Plus™ PCR Primer Set kit from Stratagene (La Jolla, CA). Cells grown to 80% confluence in tissue culture dishes were transiently transfected with the pCXN2 plasmid expressing human wild type ERβ (Addgene, Cambridge, MA) using LipofectAMINE reagent as described by the manufacturer. Gene silencing was achieved using an ERβ-specific shRNA lentiviral plasmid (pLKO.1-puro) by Sigma (St Louis, MO).

### Cell lysis, immunoprecipitation and immunoblot

Cells were extracted with NP-40 lysis buffer (1% NP-40, 150 mM NaCl, 50 mM Tris-HCl pH 8, 5 mM EDTA, 10 mM NaF, 10 mM Na_4_P_2_O_7_, 0.4 mM Na_3_VO_4_, 10 µg/ml leupeptin, 4 µg/ml pepstatin and 0.1 Unit/ml aprotinin). Cell lysates were centrifuged at 13.000 x g for 10 minutes and the supernatants were collected and assayed for protein concentration using the Bradford protein assay reagent (Bio-Rad). Proteins were separated by SDS-PAGE under reducing conditions. For co-immunoprecipitation experiments, cells were incubated with antibodies specific for EGFR for 1 hour at 4°C, then lysed and a volume equivalent to 2 mg of extracted protein for each treatment was incubated in the presence of 50 µl protein A-Sepharose beads. Following SDS-PAGE, proteins were transferred to nitrocellulose, reacted with the specific antibodies indicated and then detected with horseradish peroxidase-conjugated secondary antibodies and the chemioluminescent ECL reagent. Densitometric analysis was performed using the GS 250 Molecular Imager (Bio-Rad). For Cyclin D1 expression, cells were extracted in RIPA Buffer (1% Triton X-100, 0.1% SDS, 1% Na-deoxycholate, 150 mM NaCl, 50 mM Tris-HCl pH 7, 0.4 mM Na_3_VO_4_, 10 µg/ml leupeptin, 4 µg/ml pepstatin and 0.1 Unit/ml aprotinin) and analyzed as indicated above.

### Cell proliferation as determined by direct counting

REN or MSTO-211H cells were seeded at a density of 1×10^4^ cells/well into six-well plates in growth medium supplemented with FBS and incubated overnight at 37°C in a humidified environment containing 5% CO_2_ to allow the cells to become adherent. Cells were alternatively transiently transfected with the pCXN2 ERβ plasmid or ERβ shRNA, using the LipofectAMINE reagent as described by the manufacturer. After 24 hours cells were grown for a further 24–48 hours as indicated in complete medium or treated with 5 ng/ml of human recombinant EGF in 2% charcoal-stripped FCS growth medium. Cells were then trypsinized and stained with Trypan blue. The number of viable cells was counted in a Burker chamber within 5 min of staining. The same protocol was used to evaluate drugs sensitivity.

### Assay for Anchorage-Independent Cell Growth

Anchorage-independent growth was determined using a modification of previously described methods [40]. Briefly, a base layer of 0.6% agar in complete medium was plated in six-well plates and allowed to solidify (0.5 µg/ml Puromycin was added to wells containing shERβ transfected cells, while 250 µg/ml G418 was added to ERβ transfected cells). Next, wells were overlaid with 5×10^3^ cells per well in a 0.3% agar. A growth control well was also included with 5×10^3^ cells in medium alone (no agar) for each cell line. The plates were incubated at 37°C, 5% CO_2_ for 15 days and checked every 2 days for colony formation. At day 7, individual colonies (defined as clusters of 15 or more cells) were counted in 10 random fields.

### Confocal microscopy analysis

Immunofluorescence was performed using standard techniques. Briefly, cells were plated (1×10^5^ cells) on glass cover slips and allowed to adhere in a humidified atmosphere with 5% CO_2_ at 37°C. Wild type or transfected cells were then stimulated with EGF (5 ng/ml for 5 minutes) and subsequently fixed with 4% paraformaldehyde in PBS. Cells were permeabilized 5 minutes at RT with 0.2% Triton-X in PBS. After fixation, the cells were rinsed in PBS and incubated in a blocking solution containing 1% Gelatin and 4% bovine serum albumin (BSA) in PBS for 1 hour. Primary antibodies diluted in 2% BSA in PBS, were added in combination to the fixed cells and incubated at room temperature for 2 h. After washing in 2% BSA in PBS, the immunoreactivity was revealed using Alexa Fluor 488 goat anti-mouse IgG or tetramethylrhodamine isothiocynate (TRITC) goat anti-rabbit secondary antibodies (Invitrogen, Paisely, UK) in 2% BSA in PBS (1∶200) used separately to stain the cells for 30 min at room temperature. Negative controls were performed by substituting the primary antibodies with the 2% BSA in PBS buffer. The immuno-stained cells were rinsed with PBS and mounted in Vectashield mountant (Vector Laboratories, Burlingame, CA) containing 4′-6-Diamidino-2-phenylindole (DAPI). Confocal imaging was performed using a laser scanning LSM 510 confocal microscope (Carl Zeiss, Welwyn Garden City, UK). Alexa Fluor 488, TRITC and DAPI fluorophores were excited individually at 488 nm, 543 nm and 364 nm respectively. Single focal plain scans of 0.8 µM depth were performed at the mid diction of the cell monolayer using the 63x1.4 oil immersion objective.

### Internalization assay by flow cytometry analysis

Cell surface EGFR expression was evaluated by flow cytometry performed as described. Cells were grown in a Petri dish until confluent, washed with PBS, detached using 0.1% trypsin–EDTA and re-suspended in PBS with 1 mM CaCl2 and 1 mM MgCl2 supplemented with 4% FBS. After the indicated treatments, cells were incubated for 30 minutes at 4°C with the monoclonal anti-EGFR antibody or non-specific IgG as control, washed twice with PBS and further incubated with fluorescein isothiocynate (FITC)-conjugated goat anti-mouse secondary antibody (1∶200) for 30 minutes. After washing twice, cells were fixed with 3% paraformaldehyde in PBS at room temperature for 15 minutes and washed twice in PBS. FITC fluorescent emission was detected over the range 515–555 nm using the FL-1 detector of a FACScan flow cytometer (Becton Dickinson, Franklin Lakes, NJ) equipped with 15 mW argon–ion laser for excitation. Debris was gated out by establishing a region around the population of interest on the Forward Scatter versus Side Scatter dot plot. For each sample, 10.000 events in the region of interest were recorded at a flow rate of 200–300 cells/s. Data were processed with analysis software LYSYS II (Becton Dickinson) and are expressed as median value of EGFR expressing cells of the fluorescence histograms normalized to the corresponding negative control obtained by omitting the primary antibody.

### Statistical analysis

Statistical differences between treatment groups were measured using the one-tailed Student's test.
